# Happiness amongst Israel Defense Force (IDF) Mental Health Officers (MHO’s)

**DOI:** 10.1186/2054-314X-1-7

**Published:** 2015-03-25

**Authors:** Assaf Shelef, Calanit Zdaka, Yoram Barak

**Affiliations:** 11 Mental Health Department, Medical Corps, Israel Defense Force, Ramat-Gan, Israel; 12 Abarbanel Mental Health Center, Bat-Yam, Israel and Sackler Faculty of medicine, Tel-Aviv University, Tel-Aviv, Israel

**Keywords:** Happiness, Career, Life satisfaction

## Abstract

**Background:**

Positive psychology is the scientific study of positive experiences and positive individual traits. Happy people have a better quality of life and other benefits, including better health.

The Mental Health Department of the IDF employs a large cadre of Mental Health Officers (MHO’s). The rate of burnout among MHO’s is considered to be high.

Career satisfaction has received attention recently with publications dealing with the growing discontent of healthcare system workers. High MHO’s satisfaction is likely to result in good outcomes with patients. Continued state of dissatisfaction, may result in health problems. In this study we tried to assess levels of happiness and its correlates among MHO’s.

**Methods:**

Survey among MHO’s. Participants answered a questionnaire including the Satisfaction with Life Scale (SLS);personal details: sex, age, marital status, number of children, family income, state of healthand military details:seniority as MHO, rank, administrative executive or clinical position, unit type, army service placement.

**Results:**

In the period of the survey100 MHO’s completed the questionnaire. Amongst them were 14 psychiatrists, 25 psychologists and 60 social workers. Mean age 37.37 ± 7.12 years, mean years in army service 7.83 ± 6.47. 44% of the MHO’s were Captains, 44% Majors, 3% Lieutenant Colonels and 8% citizens working for the I.D.F.

The SLS score was analyzed in order to identify correlations to demographic and clinical variables and Pearson coefficient correlations were calculated. The mean total SLS score was 24.29 ± 5.22. The only statistically significant association with SLS score was family income (p = 0.0109).

**Conclusions:**

MHO’s reported similar levels of happiness as the mean score found in an Israeli national survey and slightly higher level of happiness compared to Israeli physicians. Family income was found to be associated to the level of happiness.

Army rank and unit type were not associated with higher satisfaction with life.

## Background

The mental health professions (psychiatrists, psychologists, social workers) have given little attention until recently to advancing optimal mental health [1].

However, in recent years a significant effort has been made in psychology to relate to questions regarding happiness, meaning of life, life satisfaction and how to develop these themes [2].

Positive psychology is the scientific study of positive experiences and positive individual traits.

Happy people have a higher quality of life.

Life satisfaction is defined as “the individual’s emotional and cognitive evaluation of their life” relating to positive emotions, lack of negative emotions and cognitive judgment regarding satisfaction and fulfillment [3].

Studies continue to identify the benefits of happiness including better health [4].

The I.D.F. Mental Health Department employs a large cadre of Mental Health Officers (MHO’s). MHO’s are psychiatrists, psychologists and social workers.

The rate of burnout among MHO’s is considered high corresponding to the rate of burnout among mental health professionals in the world, which is considered one of the highest [5]. Burnout is a very significant issue in mental health since it can lead to a decline in performance at work and worsen therapeutic outcomes [6].

In a survey among 2000 mental health workers in Italy, high levels of distress from the profession were measured in the mental health staff, detecting 1 in 5 suffered from burnout [6]. The factors which predicted burnout were: face to face interaction with patients, professional seniority, low group cohesion at the workplace and feelings of injustice [6].

Another study found that the higher the professional seniority, the lower the desire to work with patients, the feeling of success working with patients declined and the attitude towards mental illness became less humane [7].

Based on these findings there is great significance to the degree of happiness among professional health workers in general and specifically to MHO’s, since MHO’s satisfied with their work will probably provide better treatment to their patients compared to unsatisfied MHO’s. Furthermore, continued dissatisfaction can lead to health problems, as described among physicians [8]. In this study we tried to assess levels of happiness and its correlates among MHO’s.

## Methods

The participants, I.D.F Mental Health Officers, filled out an anonymous questionnaire including Satisfaction with Life Scale (SLS) developed by Diener E, et al. [9]. This short questionnaire examines the entirety of life satisfaction based on 5 statements which are:

1. In most ways my life is close to my ideal.
2. The conditions of my life are excellent.
3. I am satisfied with life.
4. So far I have gotten the important things I want in life.
5. If I could live my life over, I would change almost nothing.

These five statements are rated between 1–7:

1 = Strongly disagree

2 = Disagree

3 = Slightly disagree

4 = Neither agree or disagree

5 = Slightly agree

6 = Agree

7 = Strongly agree

The final score is the sum of the results of the five statements which reflect the degree of satisfaction with life [10]. As follows: 31–35: highly satisfied, 26–30: satisfied, 21–25: slightly satisfied, 20: Neutral, 15–19 slightly dissatisfied, 10–14: dissatisfied, 5–9 extremely dissatisfied.

In addition, the participants answered a short questionnaire regarding their personal details (sex, age, marital status, number of children, family income, and state of health; and military details: seniority as MHO, rank, administrative executive or clinical position, unit type, army service placement.

The participants’ state of health was evaluated by the question: “Do you have medical problems which have a negative impact on your satisfaction with life?”

The answer to this question was rated between 1–7, as the SLS questionnaire.

The survey was distributed during September-October 2008 via email and at professional conferences. The study was approved by the research wing of the I.D.F medical corps in the Chief medical officer’s headquarters. Helsinki committee approval was not warranted.

Statistical analysis method: the results are presentted as mean ± standard deviation. Comparison between groups was based on the non-parametric chi^2^ test. Correlation was examined using the Pearson test. Significant results were considered when p < 0.05.

## Results

In the period of the survey 100 MHO’s completed the questionnaire. Amongst them were 14 psychiatrists, 25 clinical psychologists and 60 social workers. Mean age 37.37 ± 7.12 years, mean years of professional seniority 7.83 ± 6.47. 53% were women, 47% were men. The majority of participants were married (79%), 18% single, 2% divorced, 1% widowed. The mean number of children in MHO’s families was 1.55, 27% had no children, 16% had one child, 30% had 2 children, 27% had 3 children or more.

44% of the MHO’s were Captains, 44% Majors, 3% Lieutenant Colonels and 8% citizens working for the I.D.F.

Education-20% had an undergrad academic degree, 65% had graduate degree and 14% had a doctorate.

37% of the MHO’s served in the technology and logistics division, 34% served in the ground forces, 18% served in the air force, 5% in the human resources division, 3% in the intelligence division and 2% in the navy.

17% of the HMO’s reported a family income below 10,000 NIS a month, 55% reported a family income between 10,000-20,000NIS a month and 27% reported a family income above 20,000 a month.

Where they had an administrative executive or clinical position: 10% of the MHO’s served in the headquarters and dealt with administrative executive work only, 35% dealt with clinical work only, 54% combined clinical and administrative work.

Place of service: 26% served in the mental health clinics, 22% in basic training and combat training bases, 11% served in the divisions, 14% in the headquarters, 6% in the recruiting offices, 3% in the army prison, 2% in hospitals, 16% in other units.

The majority of MHO’s (57%) reported having no medical problem impacting on their quality of life. While a minority (13%) reported having a medical problem which has an impact on their quality of life.

The objective of the SLS scale analysis was to find correlations to the demographic and clinical variables. Pearson correlation coefficients were calculated for continuous variables and the chi^2^ test was used for categorical non-parametric variables.

The SLS mean score was 24.29 ± 5.22, the range was 7–35 (see Table 1).

**Table 1:** **Satisfaction with life scale – SLS**

	Mean	Standard deviation
In most ways my life is close to my ideal.	4.72	1.29
The conditions of my life are excellent.	4.78	1.40
I am satisfied with life.	5.14	1.20
So far I have gotten the important things I want in life.	5.33	1.26
If I could live my life over, I would change almost nothing.	4.32	1.64
Sum	24.29	5.22

Legend:

Minimum score for any item = 1.

Maximum score for any item = 7.

1 = Strongly Disagree, 2 = Disagree, 3 = Slightly Disagree, 4 = neither Agree or Disagree, 5 = Slightly Agree, 6 = Agree, 7 = Strongly Agree.

**Table 2:** **Correlations with demographic and career variables**

	Chi ^2^	Pearson correlation coefficient (r)	P value
Age		0.53	0.06
Gender	0.7		0.71
Duration of service		0.15	0.14
Military Rank	3.08		0.79
Unit characterization (the mental health clinic, basic training and combat training bases, divisions, headquarters, recruiting offices, army prison, hospitals )	13.77		0.46
Job description (administrative, clinical, both)	2.41		0.65
Marital status (single, married, widowed, divorced)	3.71		0.15
Family income (all income – divided into low, middle and high income levels)	6.5		0.01

The majority of participants had a SLS score above 20 (78%).

Among all the variables, the only significant statistical correlation found was to level of family income (p = 0.01). No significant statistical correlation was found to any of the other variables (see Table 2).

## Discussion

Little is known regarding the proportion of MHO’S who are happy. No previous research has studied this subject; our research study was the first. The significance is clear: happy individuals succeed in many areas of their life including marriage, relationships, health and income. Higher achievements in the workplace have been specifically demonstrated among happy physicians [11].

Our study is interesting since rank, type of unit and type of position did not impact the degree of satisfaction with life. Furthermore, additional variables as age, sex, number of children, marital status and professional seniority did not predict the MHO’s degree of happiness.

The level of income was found to be directly correlated to the highest level of happiness. This finding was also identified in a survey by the bank of Israel in 2002 [12]. This survey included 9404 Israeli citizens over the age of 20. The majority of participants- 83% reported they are ‘satisfied’ with their life. In our survey MHO’S from various medical health professions, as a group reported similar levels of happiness as the mean score of Israeli adults, 78% of the participants in our study reported they are ‘satisfied’ or higher (according to the SLS score above 20).

MHO’s reported higher levels of happiness compared to a group of Israeli physicians. In 2007 a study examined the level of happiness of a group of 223 Israeli physicians, general physicians and psychiatrists [13]. The SLS score of these physicians was 23.6 ± 5.7

Possibly a feeling of high cohesion in an organization is a protective factor from dissatisfaction and distress.

Study limitations: the reported data were self-reports in a cross sectional format, thus evidence for casual correlation is subject to interpretation. Additionally, possibly the MHO’s who completed the survey were more cooperative and willing to contribute, traits attributed to happier and highly satisfied people. Granting this assumption stands true for data of any questionnaire survey.

## Conclusions

MHO’s reported similar levels of happiness as the mean score found in an Israeli national survey and slightly higher level of happiness compared to Israeli physicians. Family income was found associated with the level of happiness. Army rank and unit type were not associated with higher satisfaction with life.
